# Supplementary figures and images for: Behavioral, morphological, and ecological trait evolution in two clades of New World Sparrows (Aimophila and Peucaea, Passerellidae)
Source: PeerJ. 2020 Jun 19;8:e9249. doi: 10.7717/peerj.9249 (PMC7307569; doi:10.7717/peerj.9249)

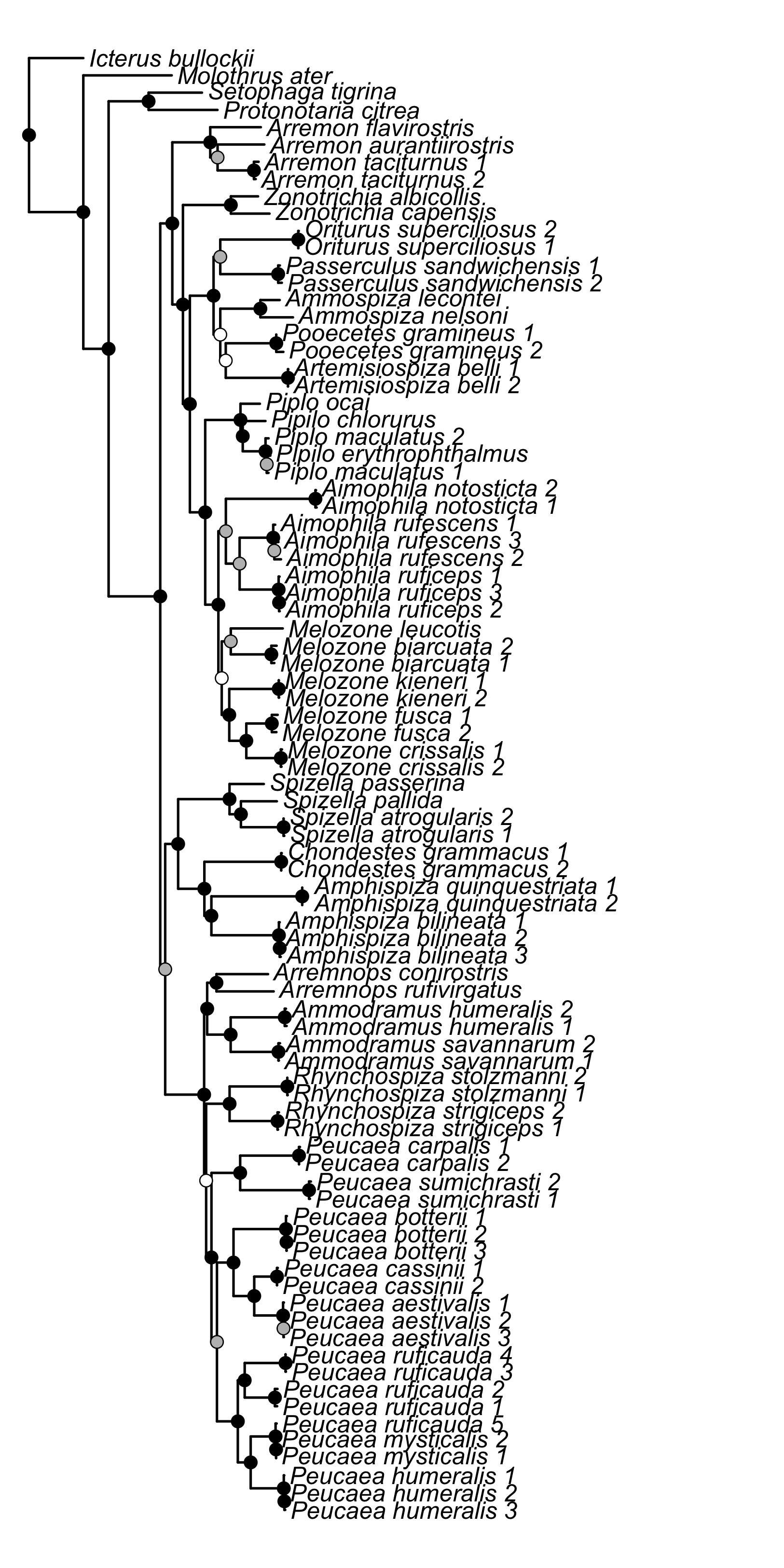

Supplement: Figure S1 — Circles on the nodes correspond to bootstrap support values, in which white circles indicate nodes that received less than 50 bootstrap support, gray indicates nodes with between 50 and 70 bootstrap support, and black indicates nodes with strong support greater than 70 bootstrap support. [file peerj-08-9249-s001.png]

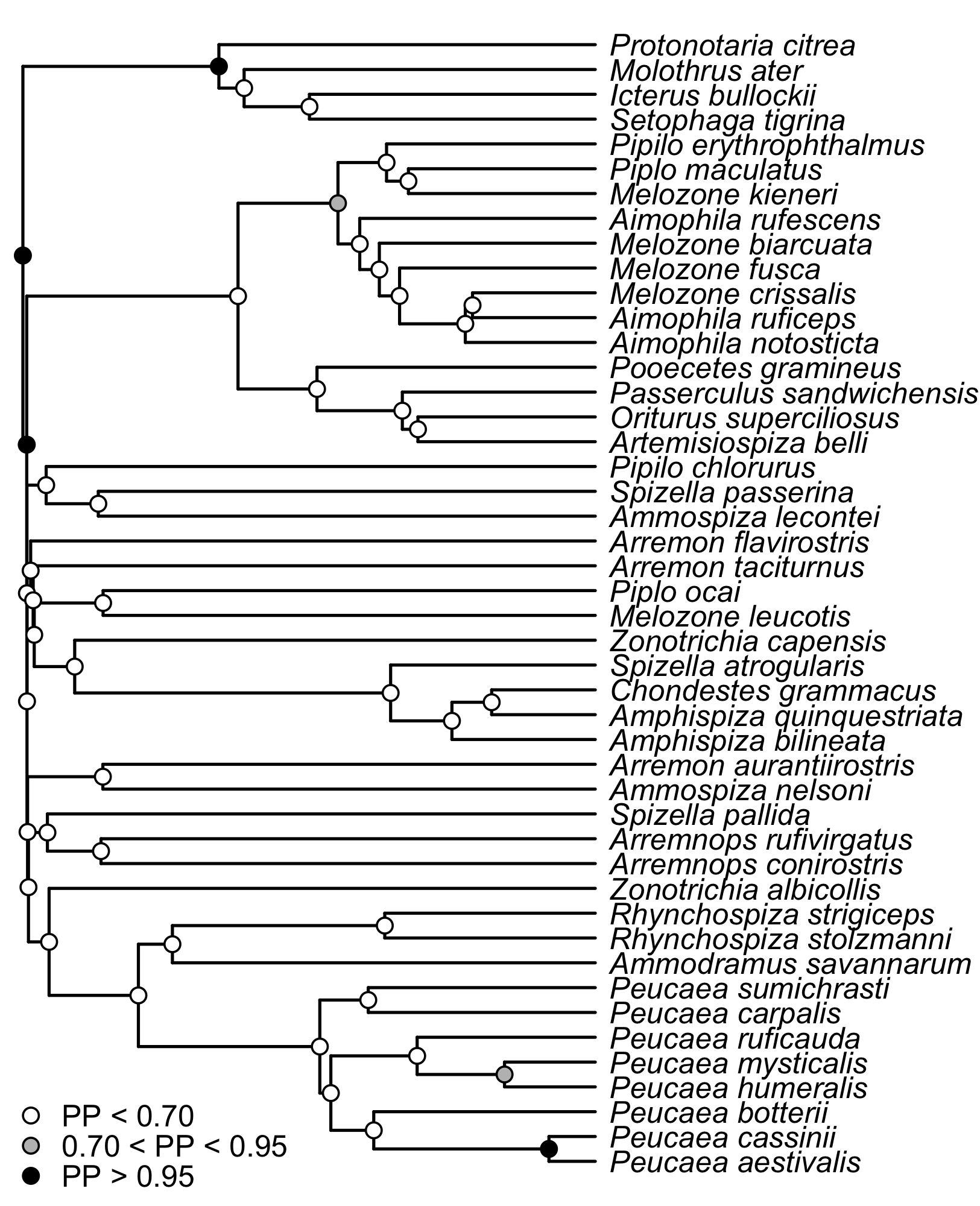

Supplement: Figure S2 — Species inferred using *BEAST. Circles on the nodes correspond to posterior probabilities, in which white circles indicate nodes with less than 70 posterior probability, gray indicates nodes with between 70 and 95 posterior probability, and black indicates nodes with strong support greater than 95 posterior probability. [file peerj-08-9249-s002.png]
