# Supplementary material for: Behavioral, morphological, and ecological trait evolution in two clades of New World Sparrows (Aimophila and Peucaea, Passerellidae)
Source: PeerJ. 2020 Jun 19;8:e9249. doi: 10.7717/peerj.9249 (PMC7307569; doi:10.7717/peerj.9249)
Supplement: Table S2 — Primer names, sequences, and sources for 4 mitochondrial and 3 nuclear gene regions analyzed in this study. [file peerj-08-9249-s004.docx]

| **Table S2:**  **Gene regions and primers used in this study.** | | | |
| --- | --- | --- | --- |
|  | Primer^1^ | Primer Sequence | Source |
|  | | | |
| **Mitochondrial (3495 bp)** | | | |
|  |  |  |  |
| Cyt *b* | L14851 | CCTACTTAGGATCATTCGCCC T | Cicero & Johnson, 2001 |
|  | L15236 | TACCTAAACAAAGAAAC[G/C/T]TG[G/A]AA | Cicero & Johnson, 2001 |
|  | L15557 | GACTGTGACAAAATCCC[G/A/T/C]TTCCA | Cicero & Johnson, 2001 |
|  | H15304 | GTAGCACCTCAGAA[C/G/T]GATATTTG | Cicero & Johnson, 2001 |
|  | H15916 | ATGAAGGGATGTTCTACTGGTTG | Cicero & Johnson, 2001 |
|  | H16065 | GGTCTTCATCT[C/T][C/T/A]GG[T/C]TTACAAGAC | Cicero & Johnson, 2001 |
|  |  |  |  |
| ND2 | L5204 | GCTAACAAAGCTATCGGGCCCAT | Cicero & Johnson, 2001 |
|  | L5494 | AATGCATGATCCACCGGCCAATGAGA | Cicero & Johnson, 2001 |
|  | L5809 | GCCTTCTCATCCATCTCCCACCTAGGATGAAT | Cicero & Johnson, 2001 |
|  | H5578 | CCTTGGAGTACTTCTGGGAATCAGA | Cicero & Johnson, 2001 |
|  | H6030 | TTGGTTAGTTCTTGGATAATGAGTCA | Cicero & Johnson, 2001 |
|  | H6312 | CTTATTTAAGGCTTTGAAGGCC | Cicero & Johnson, 2001 |
|  |  |  |  |
| COI | L7327 | CCTGCAGGAGGAGGAGA[T/C]CC | Cicero & Johnson, 2001 |
|  | H7827 | CCAGAGATTAGAGGGAATCAGTG | Cicero & Johnson, 2001 |
|  | BirdF1 | TTCTCCAACCACAAAGACATTGGCAC | Hebert et al., 2004 |
|  | BirdR1 | AGGTGGGAGATAATTCCAAATCCT | modified from Hebert et al., 2004 |
|  |  |  |  |
| ATP8 | bRus | TGGTCGAAGAAGCTTAGGTTCA | Fleischer et al., 2000 |
|  | tLys | CACCAGCACTAGCCTTTTAAG | Fleischer et al., 2000 |
|  |  |  |  |
| **Nuclear (1849 bp)** | | | |
|  |  |  |  |
| Fib5 | Fib5F | CGCCATACAGAGTATACTGTGACAT | Marini & Hackett, 2002 |
|  | Fib6R | GCCATCCTGGCGATTCTGAA | Marini & Hackett, 2002 |
|  |  |  |  |
| TGFb2 | TGFb2-5F | TTGTTACCCTCCTACAGACTTGAGTC | Sorenson et al., 2004 |
|  | TGFb2-6R | GACGCAGGCAGCAATTATCC | Sorenson et al., 2004 |
|  |  |  |  |
| RAG-1 | R50 | CTGATCTGGTAACCCCAGTGAAATCC | Irestedt et al., 2001 |
|  | R53 | TCCATGTCCTTTAAGGCACA | Irestedt et al., 2001 |

^1^ Cyt *b* (1143 bp): cytochrome *b*; ND2 (1041 bp): NADH dehydrogenase subunit 2; COI (1143 bp): cytochrome oxidase I; ATP8 (168 bp): ATPase subunit 8; Fib 5 (570 bp): β –fibrinogen intron 5; TGFb2 (628 bp): transforming growth factor *β* 2 intron 5; RAG-1 (651 bp): recombination activating protein. Letters refer to light (L) and heavy (H) strands. Numbers refer to location on the chicken (*Gallus*) sequence (Desjardins & Morais, 1990) of the 3’ end of the primer sequence. All primers are listed in the 5’ to 3’ direction. Degenerate sites are indicated by brackets.
