# Supplementary material for: Behavioral, morphological, and ecological trait evolution in two clades of New World Sparrows (Aimophila and Peucaea, Passerellidae)
Source: PeerJ. 2020 Jun 19;8:e9249. doi: 10.7717/peerj.9249 (PMC7307569; doi:10.7717/peerj.9249)
Supplement: Table S3 — List of species, character states, and sources for character state data used in this study. [file peerj-08-9249-s005.docx]

| **Table S3.**  **Species and character state data.** List of species, character states, and sources for character state data used in this study. | | | | | | | | | | | | | | | | | | | | | | | | | |
| --- | --- | --- | --- | --- | --- | --- | --- | --- | --- | --- | --- | --- | --- | --- | --- | --- | --- | --- | --- | --- | --- | --- | --- | --- | --- |
|  |  |  |  | |  | |  | |  | |  | |  | |  | |  | |  | |  | |  |  | |
| Taxon | Distribution | Range size | Habitat | Groups | | Plumage | | Postjuvenal molt | | Prenuptial  molt | | Nest | | Skull  timing | | Song | | Duetting | | Duet  type | | Source | | |  |
| *Peucaea aestivalis* | Northern Temperate | large | grassland | no | | dull | | complete | | absent | | ground | | normal | | complex | | present | | chitter | | Wolf, 1977 | | |  |
| *Peucaea cassinii* | Middle America & Northern Temperate | medium | grassland | no | | dull | | complete | | absent | | ground | | normal | | complex | | present | | chitter | | Wolf, 1977 | | |  |
| *Peucaea botterii* | Middle America & Northern Temperate | large | grassland | no | | dull | | complete | | present | | ground | | normal | | complex | | present | | chitter | | Wolf, 1977 | | |  |
| *Peucaea humeralis* | Middle America | medium | arid scrub | yes | | bright | | partial | | present | | raised | | delayed | | simple | | present | | chatter | | Wolf, 1977 | | |  |
| *Peucaea mystacalis* | Middle America | small | arid scrub | no | | bright | | partial? | | present | | ? | | delayed | | simple | | present | | chatter | | Wolf, 1977 | | |  |
| *Peucaea ruficauda* | Middle America | large | arid scrub | yes | | bright | | partial | | present | | raised | | delayed | | simple | | present | | chatter | | Wolf, 1977 | | |  |
| *Peucaea carpalis* | Middle America & Northern Temperate | medium | arid scrub | no | | dull | | partial | | present | | raised | | normal | | simple | | present | | warble | | Wolf, 1977 | | |  |
| *Peucaea sumichrasti* | Middle America | small | grassland | no | | dull | | partial | | present | | raised | | delayed | | simple | | present | | chatter | | Wolf, 1977 | | |  |
| *Ammodramus savannarum* | Middle America & Northern Temperate | large | grassland | ? | | dull | | complete | | present | | ground | | normal | | simple | | absent | | none | | Vickery, 2020 | | |  |
| *Ammodramus humeralis* | South America | large | grassland | ? | | dull | | ? | | absent | | ground | | ? | | simple | | ? | | ? | | Byers et al., 1995 | | |  |
| *Arremonops rufivirgatus* | Middle America | medium | pine-oak | no | | dull | | partial | | absent | | raised | | normal | | simple | | present | | ? | | Brush, 2020 | | |  |
| *Arremonops conirostris* | South America | large | pine-oak | ? | | dull | | ? | | ? | | ? | | ? | | simple | | ? | | ? | | Byers et al., 1995 | | |  |
| *Rhynchospiza stolzmanni* | South America | small | arid scrub | ? | | dull | | ? | | absent | | ground | | ? | | complex | | ? | | ? | | Byers et al., 1995 | | |  |
| *Rhynchospiza strigiceps* | South America | medium | arid scrub | ? | | dull | | ? | | absent | | ground | | ? | | complex | | ? | | ? | | Byers et al., 1995 | | |  |
| *Amphispiza bilineata* | Middle America & Northern Temperate | large | arid scrub & pine-oak | no | | bright | | partial | | absent | | raised | | normal | | complex | | absent | | none | | Johnson et al., 2020 | | |  |
| *Amphispiza quinquestriata* | Middle America | medium | grassland | no | | bright | | partial | | absent | | ground & raised | | normal | | complex | | absent | | none | | Wolf, 1977; Phillips & Farfan, 1993 | | |  |
| *Chondestes grammacus* | Middle America & Northern Temperate | large | pine-oak | no | | bright | | complete | | present | | ground & raised | | normal | | complex | | absent | | none | | Martin & Parrish, 2020 | | |  |
| *Spizella atrogularis* | Middle America | large | arid scrub | no | | bright | | partial | | present | | raised | | normal | | simple | | absent | | none | | Tenney, 2020 | | |  |
| *Spizella pallida* | Northern Temperate | large | pine-oak | no | | dull | | partial | | present | | raised | | normal | | simple | | absent | | none | | Grant & Knapton, 2020 | | |  |
| *Spizella passerina* | Northern Temperate | large | pine-oak | no | | dull | | partial | | present | | raised | | normal | | simple | | absent | | none | | Middleton, 2020 | | |  |
| *Aimophila notosticta* | Middle America | small | pine-oak | no | | dull | | partial | | absent | | ground | | normal | | complex | | absent | | none | | Wolf, 1977 | | |  |
| *Aimophila rufescens* | Middle America | large | pine-oak | no | | dull | | partial | | absent | | ground | | normal | | complex | | present | | squeal | | Wolf, 1977 | | |  |
| *Aimophila ruficeps* | Middle America & Northern Temperate | large | pine-oak | no | | dull | | partial | | absent | | ground | | normal | | complex | | present | | squeal | | Wolf, 1977 | | |  |
| *Melozone biarcuata* | Middle America | medium | pine-oak | ? | | bright | | ? | | absent | | raised | | ? | | simple | | present | | ? | | Byers et al., 1995; Sandoval et al., 2013 | | |  |
| *Melozone leucotis* | Northern Temperate | medium | pine-oak | ? | | bright | | ? | | absent | | ground & raised | | ? | | ? | | present | | ? | | Byers et al., 1995; Howell & Webb, 1995 | | |  |
| *Melozone kierneri* | Middle America | large | arid scrub & pine-oak | ? | | bright | | ? | | absent | | raised | | ? | | simple | | present | | squeal | | Byers et al., 1995; Wolf, 1977 | | |  |
| *Melozone crissalis* | Middle America & Northern Temperate | large | pine-oak | no | | dull | | partial | | absent | | raised | | normal | | simple | | present | | squeal | | Benedict et al., 2020 | | |  |
| *Melozone fusca* | Middle America & Northern Temperate | large | arid scrub & pine-oak | no | | dull | | partial | | absent | | raised | | normal | | simple | | present | | squeal | | Johnson & Haight, 2020 | | |  |
| *Pipilo erythropthalmus* | Northern Temperate | large | pine-oak | ? | | bright | | part | | absent | | ground | | normal | | simple | | absent | | none | | Greenlaw, 2020 | | |  |
| *Pipilo maculatus* | Middle America & Northern Temperate | large | pine-oak | no | | bright | | partial | | absent | | raised | | normal | | simple | | absent | | none | | Bartos Smith & Greenlaw, 2020 | | |  |
| *Pipilo chlorurus* | Northern Temperate | large | pine-oak | no | | bright | | partial | | present | | ground & raised | | delayed | | complex | | absent | | none | | Dobbs et al., 2020 | | |  |
| *Pipilo ocai* | Middle America | medium | pine-oak | no | | bright | | ? | | ? | | ? | | ? | | simple | | ? | |  | | Howell & Webb, 1995 | | |  |
| *Artemisiospiza belli* | Northern Temperate | large | arid scrub | no | | dull | | complete | | absent | | ground & raised | | normal | | complex | | absent | | none | | Martin & Carlson, 1998 | | |  |
| *Pooecetes gramineus* | Middle America & Northern Temperate | large | arid scrub & grassland | no | | dull | | partial | | absent | | ground | | normal | | complex | | absent | | none | | Jones & Cornely, 2020 | | |  |
| *Ammospiza nelsoni* | Northern Temperate | large | grassland | yes | | dull | | partial | | present | | ground | | normal | | simple | | absent | | none | | Shriver et al., 2020 | | |  |
| *Ammospiza lecontei* | Northern Temperate | large | grassland | no | | dull | | partial | | present | | ground | | normal | | simple | | absent | | none | | Lowther, 2020a | | |  |
| *Oriturus superciliosus* | Middle America | medium | pine-oak & grassland | yes | | dull | | ? | | absent | | ground | | ? | | simple | | ? | | ? | | Byers et al., 1995; Howell & Webb, 1995 | | |  |
| *Passerculus sandwichensis* | Middle America & Northern Temperate | large | grassland | no | | dull | | partial | | present | | ground | | ? | | simple | | absent | | none | | Wheelwright & Rising, 2020 | | |  |
| *Zonotrichia albicollis* | Northern Temperate | large | pine-oak | no | | dull | | partial | | present | | ground | | delayed | | simple | | absent | | none | | Falls & Kopachena, 2020 | | |  |
| *Zonotrichia capensis* | South America & Middle America | large | pine-oak | no | | dull | | ? | | ? | | ground & raised | | ? | | complex | | absent | | none | | Byers et al., 1995; Howell & Webb, 1995 | | |  |
| *Arremon taciturnus* | South America | large | pine-oak | no | | bright | | ? | | ? | | raised | | ? | | ? | | ? | | ? | | Hilty, 2003 | | |  |
| *Arremon aurantiirostris* | Middle America | medium | pine-oak | ? | | bright | | ? | | ? | | ground | | ? | | complex | | ? | | ? | | Howell & Webb, 1995 | | |  |
| *Arremon flavirostris* | South America | large | pine-oak | ? | | bright | | ? | | ? | | ? | | ? | | ? | | ? | | ? | | Ridgely & Tudor, 1994 | | |  |
| outgroups: |  |  |  |  | |  | |  | |  | |  | |  | |  | |  | |  | |  | | |  |
| *Icterus bullockii* | Middle America & Northern Temperate | large | pine-oak | no | | bright | | partial | | absent | | raised | | normal | | complex | | absent | | none | | Flood et al., 2020 | | |  |
| *Molothrus ater* | Northern Temperate | large | pine-oak | yes | | dull | | partial | | absent | |  | | normal | | complex | | present | | chatter | | Lowther, 2020b | | |  |
| *Protonotaria citrea* | Middle America & Northern Temperate | large | pine-oak | no | | bright | | partial | | absent | | raised | | normal | | simple | | absent | | none | | Petit, 2020 | | |  |
| *Setophaga tigrina* | Middle America & Northern Temperate | medium | pine-oak | no | | bright | | partial | | present | | raised | | normal | | simple | | absent | | none | | Baltz & Latta. 2020 | | |  |

Most information comes from sources indicated in the table. In addition, skull timing was sometimes taken from Pyle (1997) and song type from sound files obtained from the Macaulay Library of Natural Sounds, Ithaca, NY: *Peucaea aestivalis* – ML 14976, 14978, 14979, 62933, 62937, 73874, 105324, 105458, 105459, 105747; *P. botteri* – ML 14980, 14981, 14982, 14984, 14985, 40570, 45073, 45085, 45157, 112634; *P. carpalis* – ML 14986, 14989, 14990, 40573, 40574, 45072, 45151, 45178, 112636, 112637; *P. sumichrasti* – ML 15017, 15018; *P. cassinii* – ML 14991, 14992, 14993, 37724, 45040, 45043, 50231, 78368, 105220, 105542; *P. humeralis* – ML 14994, 55483, 55484, 74753; *P. mystacalis* – ML 14995; *P. ruficauda* – ML 88, 20387, 20700, 20794, 20796, 31583, 37759, 56668, 103237, 103263; *Aimophila notosticta* – ML 55485, 56639; *A. rufescens* – ML 15000, 15001, 15002, 15003, 15004, 15005, 15006, 15007, 15008, 15009; *A. ruficeps* – ML 15014, 21412, 37364, 40608, 40611, 45084, 105218, 110921, 120403, 125296; *Rhynchospiza stolzmanni* – ML 13344, 13357, 21541, 28725, 68202, 73340, 81128, 81162, 129550; *R. strigiceps* – ML 52538, 52563, 52564, 52569, 52571, 132624; *Amphispiza bilineata* – ML 15114; *A. quinquestriata* – ML 40562; *Chondestes grammacus* – ML 22963; *Spizella atrogularis* – ML 56879; *Ammodramus humeralis* – ML 17724, 20240, 34094, 67507, 67515, 104295, 117039, 120861, 121726, 132479; *A. savannarum* – ML 15105, 15106, 15108, 15110, 41980, 42242, 50245, 85190, 94379, 105523; *Melozone biarcuatum* – ML 15259, 106025; *M. kierneri* – ML 55478; *M. fusca* – ML 159, 162, 15355, 22795, 40569, 40619, 51060, 53207, 56856, 56874. *Artemisiospiza belli* – ML 42252; *Pooecetes gramineus -* ML 15362; *Oriturus superciliosus* – ML 136518; *Passerculus sandwichensis* – ML 15074; *Arremon taciturnus* – ML 127974;
